# Immunofluorescence microscopy on the blood smear identifies patients with myeloproliferative neoplasms

**DOI:** 10.1038/s41375-024-02346-z

**Published:** 2024-07-17

**Authors:** Carlo Zaninetti, Leonard Vater, Lars Kaderali, Carl C. Crodel, Tina M. Schnöder, Jessica Fuhrmann, Leonard Swensson, Jan Wesche, Carmen Freyer, Andreas Greinacher, Florian H. Heidel

**Affiliations:** 1 Institut für Transfusionsmedizin, Universitätsmedizin Greifswald, Greifswald, Germany; 2 Institut für Bioinformatik, Universitätsmedizin Greifswald, Greifswald, Germany; 3 Innere Medizin II, Abt. Hämatologie und Onkologie, Universitätsklinikum Jena, Jena, Germany; 4 Hematology, Hemostasis, Oncology and Stem Cell Transplantation, Hannover Medical School (MHH), Hannover, Germany; 5 Innere Medizin C, Universitätsmedizin Greifswald, Greifswald, Germany; 6 Leibniz Institute on Aging, Fritz-Lipmann Institute, Jena, Germany

**Keywords:** Myeloproliferative disease, Genetic testing

**To the Editor**

Myeloproliferative neoplasms (MPN) are a group of clonal stem cell disorders with heterogeneous clinical presentation [1]. Due to the risk of severe thromboembolic complications and disease progression, the early recognition of an MPN prior to the appearance of clinical complications is clearly warranted to facilitate early pharmacologic intervention [2–4]. Detection of the somatic mutations by genotyping has become an essential part of the diagnostic work-up of suspected subjects, as well as of the risk stratification after the diagnosis of MPN has been confirmed [5]. However, in many parts of the world molecular testing is barely affordable.

We have established an immunofluorescence microscopy (IF)-based method for platelet phenotyping on the peripheral blood smear [6]. This method has been proven to be highly efficient in the diagnosis of diverse hereditary platelet disorders by recognizing disease-specific changes of cell structures, including alterations of leukocytes and red blood cells (RBC) [7, 8]. Major advantages of this approach are the need of small amounts of blood (<100 μL) and the possibility to send the blood films by regular mail even long distances.

It is well-known that morphology of peripheral blood cells is also often altered in MPN [9, 10]. However, due to different methods and the heterogeneity of the patients’ populations, results are difficult to compare.

In the present study, we aimed at assessing platelet phenotype using our IF method in a cohort of patients diagnosed with MPN. The study has been registered in the German Clinical Trials Register (DRKS-ID: DRKS00032588). Three German reference centers for diagnosis and treatment of MPN took part in the study: Internal Medicine C, University Medicine Greifswald; Internal Medicine 2, University Hospital Jena; and Hematology, Hemostasis, Oncology and Stem Cell Transplantation, Hannover Medical School, Germany. The study protocol was approved by the institutional review boards of all centers. Patients or their legal guardians signed written informed consent to the investigation, which was conducted according to the Declaration of Helsinki. Healthy controls were enrolled among blood donors at the Institute for Transfusion Medicine, University Medicine Greifswald, Germany.

Blood slides were prepared using fresh EDTA-anticoagulated blood and shipped by regular mail within 5 days to the Greifswald platelet laboratory, where the analysis was performed. One blood smear was stained using the May-Grünwald-Giemsa technique, and the others labeled with primary and secondary antibodies upon fixation as previously reported [7]. The panel of used antibodies is given in the Supplementary Table 1. In detail, we investigated 13 structures including platelet surface glycoprotein IIb/IIIa and Ib/IX, three components each for platelet alpha- (von Willebrand factor, P-selectin, and thrombospondin 1) and dense granules (LAMP 1, LAMP 2, and CD63), the cytoskeletal proteins non-muscular myosin IIA (NMMIIA), filamin A, β1- and α tubulin, and the stem cell antigen CD34.

Light- and standard IF microscopy were performed using an Olympus BX40 microscope (Olympus, Hamburg, Germany) equipped with an Olympus XC10 camera, and an UplanSApo 60x immersion objective lens and the following wave length filters: WIB 460–490 nm, and WG 510–550 nm. The microscopic assessment was performed by two independent observers, who were blinded for the clinical phenotype of the MPN patients. The morphologic changes were assigned to the specific cell structure, and reported by a semiquantitative grading system as previously described [8]. The interobserver concordance was high (91%).

We enrolled 135 MPN patients and 83 healthy controls. The demographic, clinical and molecular characteristics of the enrolled subjects are provided in Table 1 and Supplementary Table 2.

**Table 1:** Clinical and morphologic features of the enrolled patients.

Pat. no.	Clinical features				Morphologic features						
	Sex/Age^a^	MPN subtype^b^	Gene carrying the driver mutation	*Additional somatic mutations*^c^*/High-molecular risk status*^d^	*Light microscopy*		*Immunofluorescence microscopy*				
					Platelet anisocytosis	RBC anisopoikilocytosis	Platelet alpha granule defect^e^	Platelet dense granule defect^f^	Platelet cytoskeleton defect^g^	Platelet surface receptor defect^h^	RBC NMMIIA aggregates^i^
1	M/73	PV	*JAK2*	No	n.a.	n.a.	Yes	Yes	No	No	No
2	F/53	MF	*CALR*	No	n.a.	n.a.	Yes	Yes	No	No	Yes
3	M/77	PV	*JAK2*	Yes/No	n.a.	n.a.	No	Yes	No	No	Yes
4	M/81	PV	*JAK2*	Yes/No	n.a.	n.a.	Yes	Yes	Yes	No	Yes
5	F/47	ET	*CALR*	Yes/No	n.a.	n.a.	Yes	Yes	Yes	No	Yes
6	F/66	ET	*JAK2*	No	n.a.	n.a.	Yes	Yes	Yes	No	Yes
7	M/55	ET	*JAK2*	No	n.a.	n.a.	Yes	Yes	No	No	Yes
8	M/36	MF	*CALR*	No	n.a.	n.a.	Yes	Yes	No	Yes	Yes
9	F/79	PV	*JAK2*	Yes/No	n.a.	n.a.	Yes	No	Yes	No	No
10	F/71	MF	*JAK2*	Yes/Yes	n.a.	n.a.	Yes	No	Yes	No	Yes
11	F/54	MPN-U	*JAK2*	No	n.a.	n.a.	No	No	No	No	Yes
12	M/44	ET	*JAK2*	No	n.a.	n.a.	No	Yes	Yes	No	Yes
13	F/79	MF	*CALR*	Yes/No	n.a.	n.a.	Yes	Yes	No	No	Yes
14	F/39	ET	*CALR*	No	n.a.	n.a.	No	No	No	No	No
15	F/61	PV	*JAK2*	Yes/No	n.a.	n.a.	Yes	No	No	No	No
16	M/59	MF	*JAK2*	No	n.a.	n.a.	Yes	Yes	Yes	No	Yes
17	M/80	PV	*JAK2*	Yes/No	n.a.	n.a.	Yes	No	Yes	No	Yes
18	M/63	ET	*JAK2*	Yes/No	n.a.	n.a.	Yes	No	No	Yes	Yes
19	M/40	PV	*JAK2*	No	n.a.	n.a.	No	No	No	No	Yes
20	F/63	ET	*JAK2*	Yes/No	n.a.	n.a.	Yes	Yes	No	No	Yes
21	F/69	MF	*JAK2*	No	n.a.	n.a.	Yes	No	No	No	Yes
22	M/67	MF	*CALR*	Yes/Yes	n.a.	n.a.	No	No	No	No	No
23	F/62	MF	*CALR*	No	n.a.	n.a.	No	Yes	No	No	Yes
24	M/78	MF	*CALR*	No	n.a.	n.a.	No	Yes	No	No	Yes
25	F/57	ET	*CALR*	Yes/No	n.a.	n.a.	No	No	No	Yes	Yes
26	M/58	PV	*JAK2*	Yes/No	n.a.	n.a.	No	No	No	No	Yes
27	F/43	MF	*JAK2*	No	n.a.	n.a.	No	Yes	No	No	No
28	M/70	PV	*JAK2*	Yes/No	n.a.	n.a.	Yes	No	No	No	Yes
29	M/79	MF	*CALR*	No	n.a.	n.a.	No	No	No	No	No
30	M/69	MF	*JAK2*	Yes/Yes	n.a.	n.a.	No	Yes	No	No	Yes
31	M/69	ET	*JAK2*	Yes/No	n.a.	n.a.	No	No	Yes	No	No
32	F/67	MF	*JAK2*	Yes/Yes	n.a.	n.a.	Yes	No	No	No	Yes
33	M/62	MF	*JAK2*	No	n.a.	n.a.	Yes	No	No	No	Yes
34	F/69	ET	*JAK2*	Yes/No	n.a.	n.a.	No	No	No	No	No
35	M/55	MF	*CALR*	n.a./n.a.	n.a.	n.a.	No	No	Yes	No	Yes
36	M/52	MF	*CALR*	No	n.a.	n.a.	No	No	No	No	Yes
37	F/68	MPN-U	TN	Yes/-	n.a.	n.a.	No	No	No	No	No
38	F/27	ET	*JAK2*	No	n.a.	n.a.	No	No	No	Yes	No
39	F/34	ET	*JAK2*	No	n.a.	n.a.	No	Yes	No	No	No
40	F/52	PV	*JAK2*	Yes/No	Yes	No	Yes	Yes	No	No	Yes
41	F/48	MF	*JAK2*	No	Yes	No	Yes	Yes	Yes	No	Yes
42	M/68	MF	*JAK2*	Yes/Yes	Yes	Yes	Yes	Yes	No	No	Yes
43	F/51	PV	*JAK2*	No	No	No	Yes	No	No	No	No
44	F/22	MF	*JAK2*	No	Yes	No	Yes	No	No	No	Yes
45	M/58	MF	*JAK2*/*MPL*	Yes/Yes	Yes	Yes	Yes	Yes	Yes	No	Yes
46	M/82	MF	*JAK2*	Yes/No	Yes	No	Yes	No	No	No	Yes
47	M/71	PV	*JAK2*	No	n.a.	n.a.	Yes	Yes	No	No	No
48	M/67	MF	*MPL*	Yes/No	Yes	Yes	Yes	No	No	No	Yes
49	M/69	PV	*JAK2*	Yes/No	Yes	No	Yes	Yes	No	No	No
50	F/64	PV	*JAK2*	No	Yes	No	No	Yes	No	No	No
51	F/52	MF	*JAK2*	No	Yes	No	Yes	No	No	No	No
52	F/55	MF	*JAK2*	Yes/No	No	Yes	Yes	No	No	No	Yes
53	M/81	PV	*JAK2*	No	n.a.	n.a.	Yes	No	No	No	Yes
54	M/75	MF	*JAK2*	No	Yes	No	No	No	No	No	Yes
55	F/68	MF	*JAK2*	Yes/No	No	No	No	No	No	No	No
56	M/48	ET	*JAK2*	Yes/No	Yes	No	Yes	No	No	No	Yes
57	M/80	MF	*JAK2*	No	Yes	Yes	Yes	Yes	No	No	Yes
58	F/62	MF	*JAK2*	Yes/Yes	Yes	Yes	Yes	No	No	No	Yes
59	F/68	MF	*CALR*	No	Yes	Yes	No	No	No	No	Yes
60	M/64	MF	*CALR*	Yes/Yes	Yes	Yes	No	No	No	No	Yes
61	F/62	PV	*JAK2*	No	Yes	No	No	No	No	No	No
62	F/59	ET	*JAK2*	No	Yes	No	No	No	No	No	No
63	F/39	MF	*MPL*	No	Yes	Yes	Yes	No	No	No	Yes
64	F/74	ET	*JAK2*	Yes/No	Yes	No	No	No	No	No	Yes
65	M/77	MPN-U	*JAK2*	Yes/-	Yes	No	Yes	Yes	No	No	Yes
66	F/53	PV	*JAK2*	Yes/No	Yes	No	No	No	No	No	Yes
67	M/77	ET	*MPL*	Yes/No	Yes	Yes	Yes	Yes	No	No	Yes
68	F/63	MF	*JAK2*	No	Yes	Yes	Yes	No	No	No	Yes
69	F/73	PV	*JAK2*	Yes/No	No	No	No	Yes	No	No	Yes
70	M/51	MF	*CALR*	Yes/No	Yes	Yes	Yes	No	No	No	Yes
71	M/64	MF	*JAK2*	Yes/No	Yes	Yes	Yes	No	No	No	Yes
72	F/67	MF	TN	Yes/Yes	Yes	Yes	No	Yes	No	No	Yes
73	M/47	MF	*MPL*	Yes/Yes	Yes	No	Yes	Yes	No	No	No
74	F/63	PV	*JAK2*	No	No	No	Yes	Yes	No	No	No
75	F/33	PV	*JAK2*	No	Yes	No	No	No	No	No	No
76	M/46	PV	*JAK2*	Yes/No	No	Yes	No	No	No	No	Yes
77	F/59	ET	*JAK2*	No	Yes	Yes	Yes	No	No	No	No
78	F/75	MF	*JAK2*	Yes/No	Yes	No	Yes	No	Yes	No	Yes
79	M/54	MF	*MPL*	No	No	No	No	No	No	No	Yes
80	F/78	MF	*CALR*	Yes/No	Yes	No	Yes	No	Yes	No	Yes
81	F/80	ET	*MPL*	No	Yes	No	Yes	No	Yes	No	Yes
82	F/31	PV	*JAK2*	No	No	No	No	No	Yes	No	Yes
83	M/60	PV	*JAK2*	Yes/No	Yes	No	No	No	Yes	No	Yes
84	M/44	ET	*CALR*	Yes/No	No	No	Yes	No	No	No	No
85	F/43	MF	*JAK2*	No	Yes	Yes	Yes	Yes	Yes	No	Yes
86	F/45	PV	*JAK2*	Yes/No	No	No	Yes	Yes	No	No	No
87	M/65	MF	*JAK2*	Yes/No	Yes	Yes	No	Yes	No	No	Yes
88	F/39	MF	*JAK2*	Yes/No	No	No	No	No	No	No	Yes
89	F/64	MPN-U	*JAK2*	Yes/-	No	No	Yes	No	No	No	Yes
90	F/39	ET	*CALR*	No	Yes	Yes	No	Yes	No	No	Yes
91	F/84	PV	*JAK2*	Yes/No	Yes	Yes	Yes	No	No	No	Yes
92	M/54	MF	*CALR*	Yes/Yes	Yes	Yes	No	No	No	No	Yes
93	F/40	ET	*JAK2*	No	Yes	Yes	Yes	Yes	Yes	No	Yes
94	F/82	MPN-U	*JAK2*	Yes/-	Yes	Yes	Yes	Yes	No	No	Yes
95	F/58	PV	*JAK2*	No	Yes	No	Yes	No	No	No	Yes
96	M/60	PV	*JAK2*	No	Yes	Yes	Yes	Yes	No	No	Yes
97	M/58	ET	*JAK2*	No	Yes	No	No	No	No	No	Yes
98	M/79	ET	*MPL*	Yes/No	Yes	Yes	Yes	Yes	No	No	Yes
99	F/76	MPN-U	TN	Yes/-	No	No	No	No	No	No	No
100	M/31	MF	TN	No	No	No	Yes	Yes	Yes	No	Yes
101	F/71	MF	*CALR*	Yes/Yes	Yes	No	No	No	No	No	No
102	M/71	ET	*JAK2*	n.a./n.a.	Yes	Yes	Yes	Yes	Yes	No	Yes
103	F/60	PV	*JAK2*	No	Yes	No	No	No	No	No	No
104	F/78	MF	*JAK2*	Yes/Yes	Yes	Yes	Yes	No	No	No	Yes
105	F/80	MF	*JAK2*	Yes/No	Yes	Yes	Yes	Yes	No	No	Yes
106	M/73	PV	*JAK2*	Yes/No	Yes	Yes	Yes	Yes	No	No	Yes
107	F/74	MF	*CALR*	No	Yes	No	Yes	Yes	No	No	Yes
108	F/43	MPN-U	*JAK2*	Yes/-	Yes	No	Yes	Yes	No	No	Yes
109	M/68	MF	*MPL*	Yes/Yes	Yes	Yes	Yes	Yes	No	No	Yes
110	M/80	PV	*JAK2*	Yes/No	No	Yes	Yes	Yes	No	No	Yes
111	M/45	PV	*JAK2*	No	Yes	No	Yes	Yes	No	No	Yes
112	M/79	MF	*JAK2*	Yes/No	n.a.	n.a.	Yes	Yes	No	No	Yes
113	M/74	MF	*JAK2*	Yes/No	Yes	No	No	Yes	No	No	No
114	F/39	ET	*JAK2*	No	n.a.	n.a.	No	Yes	No	No	No
115	F/80	MF	*JAK2*	No	Yes	Yes	Yes	Yes	No	No	Yes
116	F/67	PV	*JAK2*	No	No	No	Yes	Yes	No	No	Yes
117	F/68	ET	*JAK2*	Yes/Yes	No	Yes	Yes	Yes	No	No	Yes
118	M/50	ET	*JAK2*	Yes/No	Yes	Yes	No	Yes	No	No	Yes
119	F/71	MF	*JAK2*	Yes/Yes	No	Yes	Yes	No	No	No	Yes
120	M/75	MF	*JAK2*	No	Yes	Yes	Yes	No	Yes	No	Yes
121	F/62	MF	*CALR*	Yes/No	Yes	Yes	No	No	No	No	Yes
122	M/67	MF	*JAK2*	Yes/Yes	Yes	No	Yes	Yes	No	No	Yes
123	M/55	MF	*CALR*	No	Yes	Yes	Yes	No	Yes	No	Yes
124	F/45	PV	*JAK2*	Yes/No	Yes	No	Yes	Yes	No	No	Yes
125	F/61	MF	*JAK2*	Yes/No	Yes	No	No	No	No	No	No
126	M/28	MF	*CALR*	No	Yes	No	No	No	No	No	No
127	F/79	PV	*JAK2*	No	Yes	No	Yes	Yes	No	No	Yes
128	F/48	ET	*JAK2*	Yes/No	Yes	No	Yes	No	No	No	No
129	M/87	MPN-U	*JAK2*	Yes/-	Yes	No	Yes	No	No	No	Yes
130	F/60	MF	*JAK2*	Yes/No	Yes	Yes	Yes	Yes	Yes	Yes	Yes
131	F/47	ET	*JAK2*	Yes/No	Yes	No	No	Yes	No	No	No
132	F/61	ET	*CALR*	Yes/No	Yes	Yes	Yes	No	No	No	No
133	M/62	ET	*CALR*	Yes/No	Yes	No	No	No	No	No	Yes
134	F/44	PV	*JAK2*	Yes/No	No	No	No	No	No	No	No
135	M/70	PV	*JAK2*	n.a./n.a.	No	Yes	Yes	Yes	No	No	Yes

*Pat*. patient, *No*. number, *F* female, *M* male, *MPN* myeloproliferative neoplasm, *PV* polycythemia vera, *ET* essential thrombocythemia, *MF* primary or secondary myelofibrosis, *MPN-U* unclassifiable MPN, *TN* triple-negative, i.e., absence of mutations hitting either JAK2 or CALR or MPL; *n.a*. not available, *RBC* red blood cell, *NMMIIA* non-muscular myosin IIA.

^a^At time of investigation.

^b^According to the WHO 2016 classification [13].

^c^≥1 additional somatic mutation detected by NGS analysis of 33 genes (*ASXL1, BCOR, CBL, CEBPA, CUX1, DNMT3A, EZH2, GATA2, GNAS, GNB1, IDH1, IDH2, NF1, PHF6, PHIP, PPM1D, PRPF8, PTPN11, RAD21, RAS, RB1, RUNX1, SETBP1, SF3B1, SH2B3, SMC1A, SMC4, SRSF2, STAG2, TET2, TP53, U2AF1, ZRSR2*) with a variant allele frequency ≥ 2%.

^d^≥1 high-molecular risk mutation according to the prognostic panels of PV, ET and PMF [11, 12] - where applicable.

^e^Defined as reduced expression of at least two out of the three investigated markers of alpha granules (von Willebrand factor, P-selectin, thrombospondin 1) compared to control, as reported [8].

^f^Defined as reduced or altered expression of at least two out of the three investigated markers of lysosomes and dense granules (LAMP-1, LAMP-2, CD63) compared to control, as reported [7].

^g^Defined as altered expression of at least three out of the four investigated cytoskeletal proteins (filamin A, NMMIIA, α-tubulin, β1-tubulin) compared to control, as reported [7].

^h^Defined as reduced expression of the surface glycoprotein Ib/IX or IIb/IIIa compared to control, as reported [7];

^i^Detection of NMMIIA aggregates in RBC, as reported [8]. Pat. = patient; No. = number; F = female; M = male; MPN = myeloproliferative neoplasm; PV = polycythemia vera; ET = essential thrombocythemia; MF = primary or secondary myelofibrosis; MPN-U = unclassifiable MPN; TN = triple-negative, i.e., absence of mutations hitting either *JAK2* or *CALR* or *MPL*; n.a. = not available; RBC = red blood cell; NMMIIA = non-muscular myosin IIA.

Sixty-one of the 135 MPN patients (45%) were male, and 74 (55%) females. The median age was 63 years (range: 22–87). Thirty-six subjects (27%) had received a diagnosis of polycythemia vera; 31 (23%) of essential thrombocythemia; 60 (44%) of primary or secondary myelofibrosis; and eight (6%) of unclassifiable MPN.

Forty-two of the 83 healthy controls (51%) were male, and 41(49%) females. The median age was 36 years (range: 18–64).

By IF microscopy, we identified two frequently altered structures in MPN patients, i.e., aggregates consisting of NMMIIA in the RBC and altered expression of platelet alpha granules. RBC aggregates of NMMIIA were found in 98 (73%) of the MPN patients (Fig. 1A). In 68 of these subjects (69%), this finding was accompanied by reduced expression of at least two out of the three investigated platelet alpha granule markers (Fig. 1B). Fourteen (10%) MPN subjects showed solely reduced expression of at least two alpha granule markers. Only 16 (12%) MPN patients displayed normal findings. In strong contrast, none of the controls showed either NMMIIA aggregates in the RBC or alterations of more than one platelet alpha granule marker. The other investigated structures were substantially non-informative as only 7 MPN patients (5%) displayed alterations assignable to platelet cytoskeleton and/or dense granules. Of the MPN patients with altered phenotype by IF microscopy, 41 (52%) and 62 (78%) of the 79 evaluable subjects displayed by light microscopy a remarkable RBC anisopoikilocytosis and platelet anisocytosis, respectively. Except for a leuko-erythroblastic picture, which was detectable in in 25/79 subjects (32%), no major alterations of the RBC or white blood cells were apparent.

**Fig. 1:**
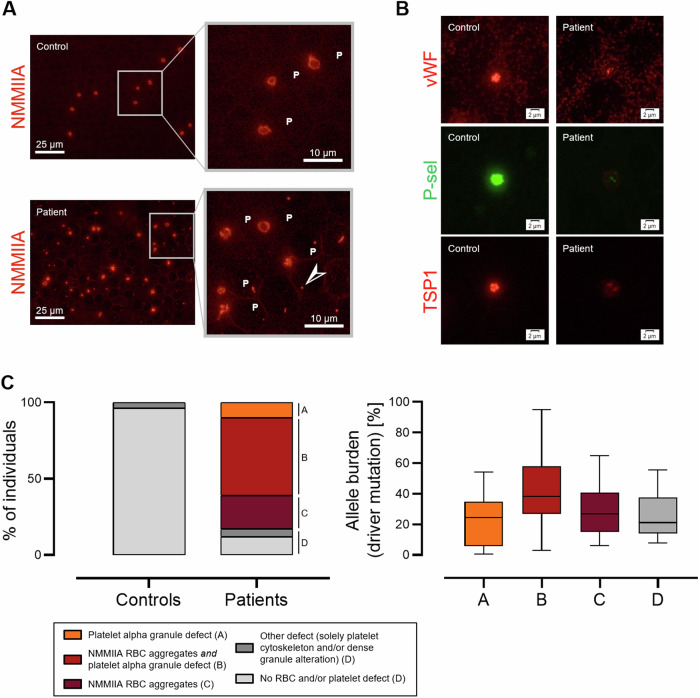
Morphological changes of platelets and red blood cells (RBC) in 135 patients with myeloproliferative neoplasms (MPN) and 83 healthy controls by immunofluorescence microscopy on the blood smear. **A** Representative picture of aggregates of non-muscular myosin IIA (NMMIIA) in RBC of a MPN patient in comparison to a healthy control. P indicates platelets, arrows indicate the RBC aggregates. **B** Reduced expression of the alpha granule markers von Willebrand factor (vWF), P-selectin (P-sel) and thrombospondin 1 (TSP1) in a MPN patient compared to a healthy control. **C** Left. Schematic representation of the spectrum of morphologic alterations found in patients and controls, and their prevalence. Right. Allele burden of mutations in *JAK2*, *CALR* or *MPL* gene within MPN patients grouped according to the found morphologic change(s), which are designated by capital letters at the stacked bar graph. Boxes represent the interquartile range, bars within the boxes designate the median values, and whiskers extend to the range of data (minimum and maximum).

We found morphologic changes of peripheral RBC and platelets, which clearly differentiated MPN patients from the healthy controls. Is interesting to note that the 68 MPN patients with concomitantly altered platelet- *and* RBC phenotype showed the highest median allele burden of their MPN driver mutations hitting *JAK2-*, *CALR-* or *MPL* gene (Table 1), whereas those with none of these morphological alterations had the lowest (Fig. 1C). The subgroup of patients with morphological changes in both platelets *and* RBC was also featured by the highest prevalence of individuals carrying additional, non-driver mutations overall (61%) as well as including at least one variant considered at-high-risk of progression (15%) [11, 12] (Supplementary Fig. 1). This suggests a possible correlation between the morphologic phenotype and other clinical features of MPN, which might be relevant also for the prognosis.

Aggregates of NMMIIA in the RBC are also typical for two hereditary platelet disorders associated with constitutional dyserythropoiesis due to germline mutations in the transcription regulator *GATA1* or *GFI1B* [8], and might represent a novel marker of dyserythropoieis in the peripheral blood. This may link the pathogenesis of somatic mutations in MPN and germline mutations in hereditary platelet disorders [7, 10].

Further studies are required to investigate this aspect as well as to identify mechanisms leading to the morphologic changes found in platelets and RBC of MPN subjects.

In conclusion, blood cell phenotyping by IF on the peripheral blood smear seems to be a clinically useful and easy-to-apply additional diagnostic tool to identify patients with MPN. This approach might be particularly useful in low- and middle-income countries with limited access to second-level diagnostic tools to stratify patients who may benefit from further genetic testing, as well in high-income country as Germany as a screening tool prior to next generation sequencing.

### Supplementary information

Supplementary material

## Data Availability

Inquiries about data access should be made to the corresponding author.
