# Supplementary material for: Immunofluorescence microscopy on the blood smear identifies patients with myeloproliferative neoplasms
Source: Leukemia. 2024 Jul 17;38(9):2051–8. doi: 10.1038/s41375-024-02346-z (PMC11347374; doi:10.1038/s41375-024-02346-z)
Supplement: Supplementary file 1 — Supplementary material [file 41375_2024_2346_MOESM1_ESM.docx]

**Immunofluorescence microscopy on the blood smear identifies patients with myeloproliferative neoplasms**

Carlo Zaninetti, et al.

**SUPPLEMENTARY INFORMATION**

**Suppl. Table 1. Antibodies used for the immunofluorescence microscopy assessment of the enrolled subjects**

| **Marker-protein** | **Host** | **Final dilution** | **Company, code** |
| --- | --- | --- | --- |
| *Primary antibodies* |  |  |  |
| MYH9 | Rabbit | 1:10000 | Sigma Aldrich, M8064 |
| β1 Tubulin | Mouse | 1:200 | Sigma Aldrich, T4026 |
| α tubulin | Mouse | 1:2000 | Sigma Aldrich, DM1A |
| Filamin A | Mouse | 1:4000 | Santa Cruz Biotechnology, sc58764 |
| Thrombospondin | Rabbit | 1:400 | Abcam, ab85762 |
| P-Selectin | Mouse | 1:400 | BD Biosciences, BD 555522 |
| Von Willebrand Factor | Rabbit | 1:2000 | DAKO, A0082 |
| LAMP 1 | Mouse | 1:20 | Santa Cruz Biotechnology, sc18821 |
| LAMP 2 | Mouse | 1:20 | Santa Cruz Biotechnology, sc18822 |
| CD63 | Mouse | 1:5000 | BD Biosciences, BD 556019 |
| GPIb/IX | Mouse | 1:500 | Bio-Rad (AbD Serotec), MCA594 |
| GPIIb/IIIa | Mouse | 1:2000 | Beckman Coulter, PN IM0145 |
| CD34 | Mouse | 1:100 | ImmunoLogic, ILM1343-C05 |
| Glycophorin A | Mouse | 1:100 | Proteintech Group Inc, 66778-1-Ig |
| *Secondary antibodies* |  |  |  |
| ALEXAFluor 568* | Goat | 1:400 | Invitrogen, A11011 |
| ALEXAFluor 488* | Goat | 1:400 | Invitrogen, A11001 |

**Suppl. Table 2. Demographic and morphologic features of the enrolled healthy controls**

| **Sub.**  **no.** | **Sex/Age^*^** | **Morphologic features** | | | | | | |
| --- | --- | --- | --- | --- | --- | --- | --- | --- |
|  |  | ***Light microscopy*** | | ***Immunofluorescence microscopy*** | | | | |
|  |  | **Platelet anisocytosis** | **RBC anisopoikilocytosis** | **Platelet alpha granule defect^‡^** | **Platelet dense granule defect^§^** | **Platelet cytoskeleton defect^¶^** | **Platelet surface receptor defect^#^** | **RBC NMMIIA aggregates^**^** |
| 1 | M/37 | No | No | No | Yes | No | No | No |
| 2 | M/24 | No | No | No | No | No | No | No |
| 3 | M/41 | No | No | No | Yes | No | No | No |
| 4 | F/64 | No | No | No | No | No | No | No |
| 5 | F/25 | No | No | No | No | No | No | No |
| 6 | M/39 | No | No | No | No | No | No | No |
| 7 | M/39 | No | No | No | No | No | No | No |
| 8 | F/26 | No | No | No | Yes | No | No | No |
| 9 | M/36 | No | No | No | No | No | No | No |
| 10 | M/28 | No | No | No | No | No | No | No |
| 11 | F/24 | No | No | No | No | No | No | No |
| 12 | F/51 | No | No | No | No | No | No | No |
| 13 | F/43 | No | No | No | No | No | No | No |
| 14 | F/54 | No | No | No | No | No | No | No |
| 15 | F/36 | No | No | No | No | No | No | No |
| 16 | M/59 | No | No | No | No | No | No | No |
| 17 | M/25 | No | No | No | No | No | No | No |
| 18 | F/62 | No | No | No | No | No | No | No |
| 19 | M/34 | No | No | No | No | No | No | No |
| 20 | F/57 | No | No | No | No | No | No | No |
| 21 | M/33 | No | No | No | No | No | No | No |
| 22 | M/20 | No | No | No | No | No | No | No |
| 23 | M/45 | No | No | No | No | No | No | No |
| 24 | M/26 | No | No | No | No | No | No | No |
| 25 | F/32 | No | No | No | No | No | No | No |
| 26 | M/32 | No | No | No | No | No | No | No |
| 27 | F/34 | No | No | No | No | No | No | No |
| 28 | M/55 | No | No | No | No | No | No | No |
| 29 | M/23 | No | No | No | No | No | No | No |
| 30 | M/36 | No | No | No | No | No | No | No |
| 31 | F/35 | No | No | No | No | No | No | No |
| 32 | M/46 | No | No | No | No | No | No | No |
| 33 | M/34 | No | No | No | No | No | No | No |
| 34 | F/53 | No | No | No | No | No | No | No |
| 35 | M/37 | No | No | No | No | No | No | No |
| 36 | M/37 | No | No | No | No | No | No | No |
| 37 | M/44 | No | No | No | No | No | No | No |
| 38 | M/55 | No | No | No | No | No | No | No |
| 39 | F/45 | No | No | No | No | No | No | No |
| 40 | M/56 | No | No | No | No | No | No | No |
| 41 | M/32 | No | No | No | No | No | No | No |
| 42 | F/45 | No | No | No | No | No | No | No |
| 43 | M/22 | No | No | No | No | No | No | No |
| 44 | F/43 | No | No | No | No | No | No | No |
| 45 | F/53 | No | No | No | No | No | No | No |
| 46 | F/35 | No | No | No | No | No | No | No |
| 47 | F/54 | No | No | No | No | No | No | No |
| 48 | F/24 | No | No | No | No | No | No | No |
| 49 | F/22 | No | No | No | No | No | No | No |
| 50 | F/26 | No | No | No | No | No | No | No |
| 51 | F/28 | No | No | No | No | No | No | No |
| 52 | M/29 | No | No | No | No | No | No | No |
| 53 | F/55 | No | No | No | No | No | No | No |
| 54 | M/24 | No | No | No | No | No | No | No |
| 55 | F/60 | No | No | No | No | No | No | No |
| 56 | M/56 | No | No | No | No | No | No | No |
| 57 | F/26 | No | No | No | No | No | No | No |
| 58 | F/50 | No | No | No | No | No | No | No |
| 59 | F/36 | No | No | No | No | No | No | No |
| 60 | F/24 | No | No | No | No | No | No | No |
| 61 | F/18 | No | No | No | No | No | No | No |
| 62 | M/50 | No | No | No | No | No | No | No |
| 63 | M/20 | No | No | No | No | No | No | No |
| 64 | M/29 | No | No | No | No | No | No | No |
| 65 | M/59 | No | No | No | No | No | No | No |
| 66 | F/24 | No | No | No | No | No | No | No |
| 67 | F/37 | No | No | No | No | No | No | No |
| 68 | M/54 | No | No | No | No | No | No | No |
| 69 | M/49 | No | No | No | No | No | No | No |
| 70 | F/44 | No | No | No | No | No | No | No |
| 71 | M/22 | No | No | No | No | No | No | No |
| 72 | F/27 | No | No | No | No | No | No | No |
| 73 | F/39 | No | No | No | No | No | No | No |
| 74 | M/46 | No | No | No | No | No | No | No |
| 75 | F/21 | No | No | No | No | No | No | No |
| 76 | F/49 | No | No | No | No | No | No | No |
| 77 | M/47 | No | No | No | No | No | No | No |
| 78 | F/35 | No | No | No | No | No | No | No |
| 79 | F/28 | No | No | No | No | No | No | No |
| 80 | M/25 | No | No | No | No | No | No | No |
| 81 | M/53 | No | No | No | No | No | No | No |
| 82 | M/30 | No | No | No | No | No | No | No |
| 83 | F/41 | No | No | No | No | No | No | No |

Legend. ^*^ at time of investigation; ^‡^ defined as reduced expression of at least two out of the three investigated markers of alpha granules (von Willebrand factor, P-selectin, thrombospondin 1) compared to control as reported [1]; ^§^ defined as reduced or altered expression of at least two out of the three investigated markers of lysosomes and dense granules (LAMP-1, LAMP-2, CD63) compared to control as reported [2]; ^¶^ defined as altered distribution of at least three out of the four investigated cytoskeletal markers (filamin A, NMMIIA, α-tubulin, β1-tubulin) compared to control as reported [2]; ^#^ defined as reduced expression of the surface glycoprotein Ib/IX or IIb/IIIa compared to control as reported [2]; ^**^ detection of NMMIIA aggregates in RBC as reported [1].
Abbreviations. Sub. = subject; No. = number; F = female; M = male; RBC = red blood cell; NMMIIA = non-muscular myosin IIA.

**Suppl. Figure 1. Prevalence spectrum of non-driver mutation overall^‡^ and at high-molecular-risk^§^ in MPN patients grouped according to the found morphologic alteration(s)**

**
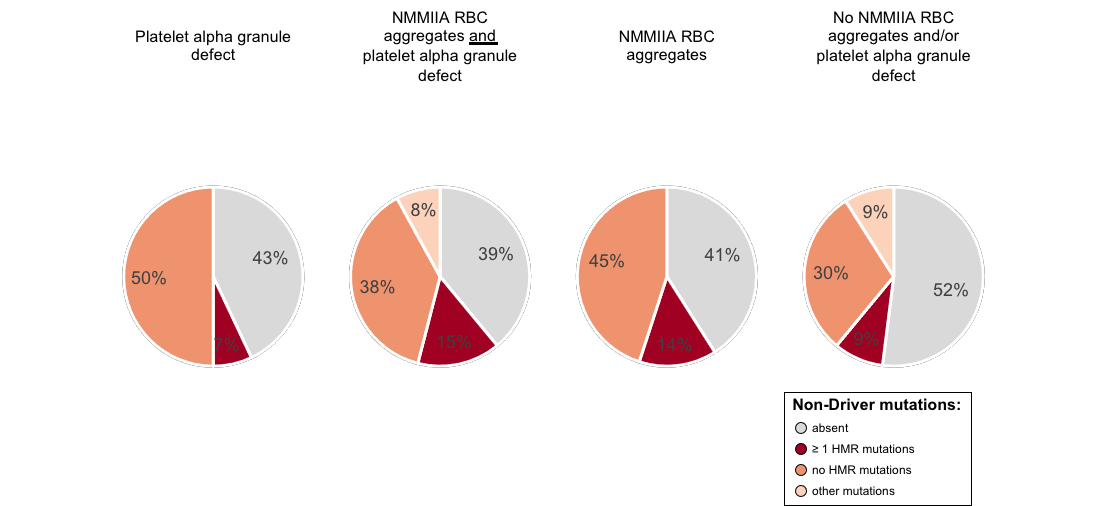
**

Legend. ^‡^ ≥ 1 additional somatic mutation detected by NGS analysis of 33 genes (*ASXL1, BCOR, CBL, CEBPA, CUX1, DNMT3A, EZH2, GATA2, GNAS, GNB1, IDH1, IDH2, NF1, PHF6, PHIP, PPM1D, PRPF8, PTPN11, RAD21, RAS, RB1, RUNX1, SETBP1, SF3B1, SH2B3, SMC1A, SMC4, SRSF2, STAG2, TET2, TP53, U2AF1, ZRSR2*) with a variant allele frequency ≥ 2%; ^§^ ≥ 1 high-molecular risk mutation according to the prognostic panels of polycythemia vera, essential thrombocythemia and primary myelofibrosis [3,4] - where applicable. Abbreviation. HMR = high-molecular risk mutations.

**References**

1. Zaninetti C, Rivera J, Vater L, Ohlenforst S, Leinøe E, Böckelmann D, et al. Aggregates of nonmuscular myosin IIA in erythrocytes associate with GATA1- and GFI1B-related thrombocytopenia. J Thromb Haemost. 2024; 22(4):1179-1186.
2. Zaninetti C, Leinøe E, Lozano ML, Rossing M, Bastida JM, Zetterberg E, et al. Validation of immunofluorescence analysis of blood smears in patients with inherited platelet disorders. J Thromb Haemost. 2023; 21(4):1010-1019.
3. Tefferi A, Lasho TL, Guglielmelli P, Finke CM, Rotunno G, Elala Y, et al. Targeted deep sequencing in polycythemia vera and essential thrombocythemia. Blood Adv 2016; 1(1):21-30.
4. Guglielmelli P, Lasho TL, Rotunno G, Score J, Mannarelli C, Pancrazzi A, et al. The number of prognostically detrimental mutations and prognosis in primary myelofibrosis: an international study of 797 patients. Leukemia. 2014; 28(9):1804-1810.
